# Acute Abdominal Aortic Occlusion in a COVID-19 Patient Despite Early Anticoagulation: A Case Report

**DOI:** 10.7759/cureus.91263

**Published:** 2025-08-29

**Authors:** Taichi Fujimori, Ryuichi Ohta, Chiaki Sano

**Affiliations:** 1 Community Care, Unnan City Hospital, Unnan, JPN; 2 Community Medicine Management, Shimane University Faculty of Medicine, Izumo, JPN

**Keywords:** acute aortic occlusion, anticoagulation, arterial thrombosis, covid-19, critical limb ischemia, elderly patients, hypercoagulability

## Abstract

Thromboembolic events related to coagulopathy have been frequently reported in patients with coronavirus disease 2019 (COVID-19), with elevated D-dimer levels being a significant risk factor for these events. While arterial thrombosis is less common than venous thrombosis, there are reports of myocardial infarction, ischemic stroke, and acute limb ischemia. Complete abdominal aortic occlusion, however, is exceptionally rare. We present a rare case of a 94-year-old male who developed complete abdominal aortic occlusion on day four of COVID-19 illness, despite early initiation of prophylactic anticoagulation along with standard antiviral and steroid therapy. The patient presented with a markedly elevated D-dimer level. After the sudden onset of severe pain from the back to the lower limbs, paralysis, and coldness of both legs, contrast-enhanced CT revealed thrombotic occlusion extending from the abdominal aorta to both iliac arteries. This case suggests that COVID-19-associated coagulopathy may progress to a severe state unmanageable by anticoagulation alone and underscores the importance of rapid evaluation and intervention for arterial thrombosis, including abdominal aortic occlusion, when acute limb ischemia-like symptoms occur.

## Introduction

COVID-19 is primarily a respiratory viral infection but is known to be associated with systemic inflammation and coagulopathy, frequently leading to thromboembolic complications [1]. In particular, markedly elevated D-dimer levels are considered a key indicator of thrombosis risk, and numerous cases of venous thromboembolism, such as deep vein thrombosis and pulmonary embolism, have been reported [2].

In contrast, arterial thrombotic events associated with COVID-19 are less frequently reported. However, severe and life-threatening complications, such as acute myocardial infarction, ischemic stroke, acute limb ischemia, and aortic occlusion, have been observed, necessitating early recognition and management [3]. The underlying pathophysiology is thought to involve multifactorial mechanisms based on Virchow's triad as follows: endothelial injury caused by viral infection, cytokine storm, platelet activation, and circulatory stasis. Imaging studies, especially contrast-enhanced CT, are useful for diagnosing such arterial occlusions. The mainstay of treatment is anticoagulation therapy, with thrombus removal or surgical intervention performed as needed [4].

Reports of abdominal aortic occlusion are exceptionally rare, particularly during the acute phase of COVID-19. Only a few cases have been reported to date, including a case series of eight patients with aortic floating thrombus, an individual report of acute aortic thrombosis, and a review of published cases in the context of COVID-19 [5-7]. Such cases often carry a high risk of morbidity and mortality due to delayed recognition and limited treatment options. We report a case of a 94-year-old male who developed complete abdominal aortic occlusion early in the course of COVID-19 despite receiving prophylactic anticoagulation. From admission, the patient had elevated D-dimer levels and was started on subcutaneous heparin calcium, dexamethasone, and remdesivir. On the early morning of day four, he developed sudden severe pain extending from his back to both lower limbs, paralysis, absent bilateral femoral pulses, and marked coldness. Contrast-enhanced CT revealed thrombotic occlusion extending from the abdominal aorta to the iliac arteries. Despite prophylactic anticoagulation, a fatal arterial thrombotic event occurred, highlighting the unpredictable nature of COVID-19-associated thrombosis and the limitations of current management strategies. This case offers valuable insights for future therapeutic approaches.

## Case presentation

A 94-year-old man, who had been largely independent in his activities of daily living with minimal assistance (certified as requiring support level 1 under Japan’s long-term care insurance system), with a history of atrioventricular block requiring permanent pacemaker implantation, presented to our secondary care hospital with general malaise during the acute phase of COVID-19. Although he was a non-smoker at the time, he had a smoking history dating back about 50 years. Six days prior to admission, he experienced a transient episode of weakness resembling syncope. He visited a higher-level medical facility, where a pacemaker check revealed a recent onset of atrial fibrillation and ventricular pacing since approximately eight days prior. The episode was attributed to hypotension due to the new arrhythmia. Given the patient's extreme age and brief duration of atrial fibrillation, anticoagulation was not initiated. These events occurred before the COVID-19 diagnosis without respiratory symptoms, making a direct association unlikely.

**Figure 1:**
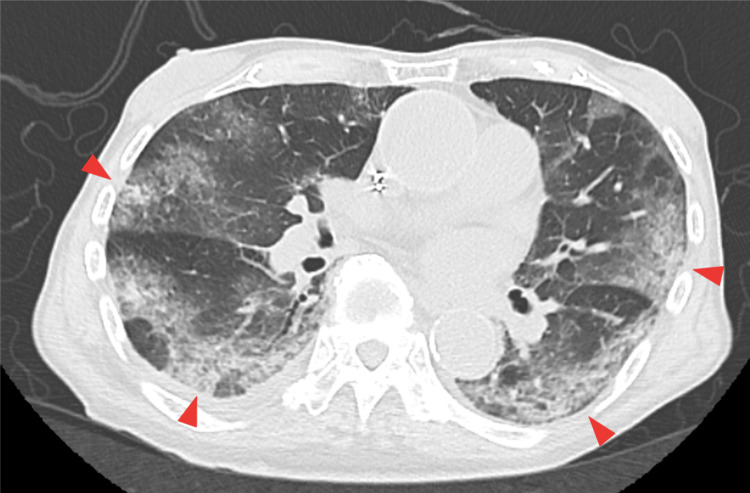
Bilateral ground-glass opacities and consolidations on admission chest CT. Non-contrast axial chest CT on the day of admission showing bilateral ground-glass opacities and peripheral consolidations (arrowheads), consistent with COVID-19 pneumonia. These findings correspond to a CO-RADS score of 5, indicating a very high level of suspicion for COVID-19, consistent with the positive antigen test result. The lesions are predominantly distributed in the posterior and peripheral regions of both lungs, demonstrating a typical radiological pattern of SARS-CoV-2 infection. These findings supported the clinical diagnosis and guided the initiation of antiviral and corticosteroid therapy. CO-RADS: COVID-19 Reporting and Data System

Four days prior to admission, his condition began to worsen with dyspnea, likely reflecting the progression of COVID-19 pneumonia. On the day of admission, despite having an appetite, he felt unwell and visited his primary care physician. Chest radiography revealed suspected pneumonia, prompting referral to our hospital. Upon presentation, he was febrile and produced copious sputum, a symptom reportedly common for him, but he had no cough, nasal discharge, or nausea. Further evaluation led to a diagnosis of COVID-19 pneumonia affecting both lungs, confirmed by a positive SARS-CoV-2 antigen qualitative test (Figure 1). Remdesivir and dexamethasone were initiated, and given the markedly elevated D-dimer level (53.60 μg/mL), prophylactic anticoagulation with subcutaneous heparin calcium was started.

His past medical history included sinusitis, pacemaker implantation for complete atrioventricular block, glaucoma, anemia, pruritus, and restless legs syndrome. His medication list included carbocisteine 500 mg three times daily, rebamipide 100 mg three times daily, clarithromycin 200 mg once daily, loratadine 10 mg once daily, polaprezinc orally disintegrating tablet 75 mg twice daily, pramipexole 0.125 mg at bedtime, and Foliamin 5 mg once daily. In addition, he used carteolol hydrochloride eye drops (2%) once daily in the right eye. Most of these medications had been taken on a chronic basis prior to admission, although the precise duration of each drug was not available.

Physical examination

On arrival, he was alert and oriented to person, place, and date. Vital signs were as follows: temperature 37.3℃, blood pressure 200/92 mmHg, heart rate 76 bpm, respiratory rate 16 breaths/min, and peripheral oxygen saturation (SpO₂) 93% with oxygen via nasal cannula at 2 L/min. The jugular venous pressure was slightly elevated, but no abnormal heart sounds were detected. Fine crackles were heard in both lungs, predominantly in the lower lung fields. There was no edema in the lower legs, and dorsalis pedis pulses were palpable bilaterally.

Laboratory findings and diagnosis

Initial blood tests revealed elevated liver enzymes, aspartate aminotransferase (AST): 1,327 IU/L, alanine aminotransferase (ALT): 831 IU/L, high lactate dehydrogenase (LDH): 1,093 IU/L, and evidence of acute liver injury associated with COVID-19. Renal function was mildly impaired with blood urea nitrogen (BUN) 42.3 mg/dL and creatinine 1.13 mg/dL (estimated glomerular filtration rate {eGFR}: 46.0 mL/min/1.73 m²). Inflammatory markers were moderately elevated (C-reactive protein {CRP}: 7.14 mg/dL), and creatine kinase (CK) was mildly increased (294 IU/L). Arterial blood gas analysis (temperature-corrected, oxygen via nasal cannula) showed pH 7.470, pO₂ 80.5 mmHg, pCO₂ 30.2 mmHg, HCO₃⁻ 22.0 mmol/L, base excess (BE) -0.7, and lactate 1.5 mmol/L, without evidence of metabolic acidosis or hyperlactatemia. B-type natriuretic peptide (BNP) was 598.9 pg/mL, and troponin I was 0.112 ng/mL. Given the elevated blood pressure, systemic inflammation due to COVID-19, and suspected hypertensive heart failure, intravenous nicardipine was administered for blood pressure control. COVID-19 treatment consisted of intravenous dexamethasone (6.6 mg/day) and remdesivir (200 mg on day one, followed by 100 mg daily). Given the markedly elevated D-dimer level (53.60 μg/mL), subcutaneous heparin calcium (5,000 IU twice daily) was initiated as prophylactic anticoagulation.

On day four of hospitalization, the patient developed signs of systemic hypoperfusion, including elevated lactate (4.0 mmol/L), worsening metabolic acidosis, and markedly increased CK (3207 IU/L) and LDH (751 IU/L), suggestive of acute muscle injury and prerenal azotemia. The laboratory data at that time are summarized in Table 1. Based on these findings, acute arterial ischemia was suspected, and contrast-enhanced CT was performed.

**Table 1:** Laboratory data on day four of hospitalization. Laboratory data on day four of hospitalization, at the time of acute arterial ischemia. The results show elevated liver enzymes, markers of muscle injury, renal dysfunction, and a prolonged prothrombin time that may reflect early coagulation abnormalities. D-dimer levels were markedly elevated at 53.60 µg/mL on admission, highlighting the hypercoagulable state. It was not re-measured on the day of thrombotic occlusion, and therefore is described here rather than included in Table 1.

Markers	Levels	Reference range
White blood cells	14.5×10^3^/μL	3.5-9.1×10^3^/μL
Neutrophils	89.8%	44.0-72.0%
Lymphocytes	2.7%	18.0-59.0%
Hemoglobin	13.5 g/dL	11.3-15.2 g/dL
Hematocrit	38.9%	33.4-44.9%
Mean corpuscular volume	87.7 fL	79.0-100.0 fL
Platelets	19.4×10^4^/μL	13.0-36.9×10^4^/μL
Total bilirubin	1.3 mg/dL	0.2-1.2 mg/dL
Aspartate aminotransferase	154 IU/L	8-38 IU/L
Alanine aminotransferase	362 IU/L	4-43 IU/L
Lactate dehydrogenase	751 IU/L	121-245 U/L
Creatine kinase	3,207 IU/L	59-248 IU/L
Blood urea nitrogen	40.9 mg/dL	8-20 mg/dL
Creatinine	0.80 mg/dL	0.40-1.10 mg/dL
Serum Na	148 mEq/L	135-150 mEq/L
Serum K	4.1 mEq/L	3.5-5.3 mEq/L
Serum Cl	116 mEq/L	98-110 mEq/L
PT (%)	42.9%	70-130%
Prothrombin time - international normalized ratio (PT-INR)	1.56	NA
Activated partial thromboplastin time (APTT)	36.7 s	25.0-40.0 s

Clinical course

**Figure 2:**
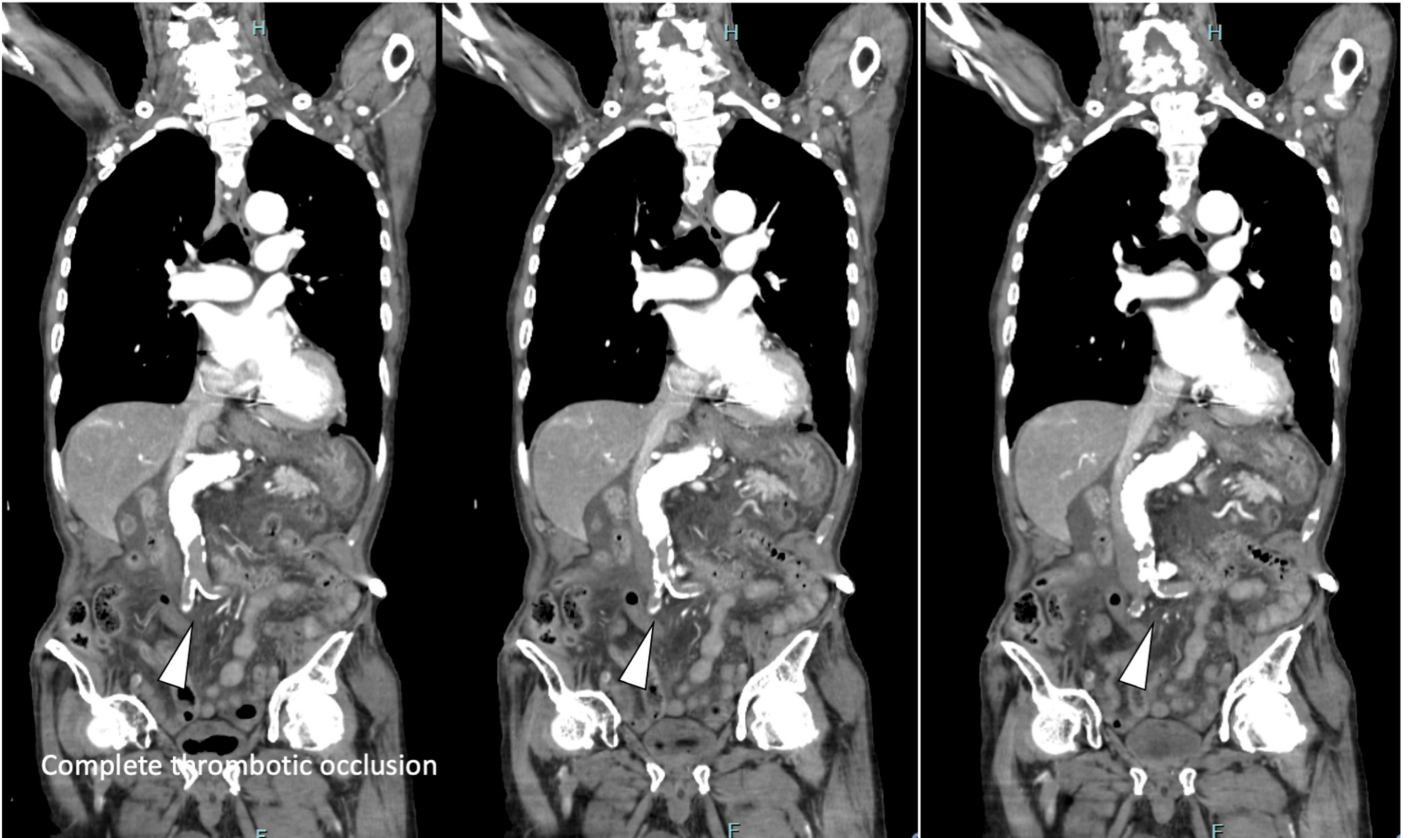
Arterial phase coronal CT images showing complete abdominal aortic occlusion extending to the common iliac arteries. Serial contrast-enhanced coronal CT images of the abdomen in the arterial phase on day four of hospitalization showing complete thrombotic occlusion of the abdominal aorta extending to the bilateral common iliac arteries (arrowheads). No contrast enhancement is observed distal to the level of occlusion, indicating the absence of arterial blood flow. These findings in the arterial phase are consistent with acute thrombotic occlusion of the abdominal aorta and explain the patient's abrupt bilateral lower limb paralysis and absent femoral pulses.

**Figure 3:**
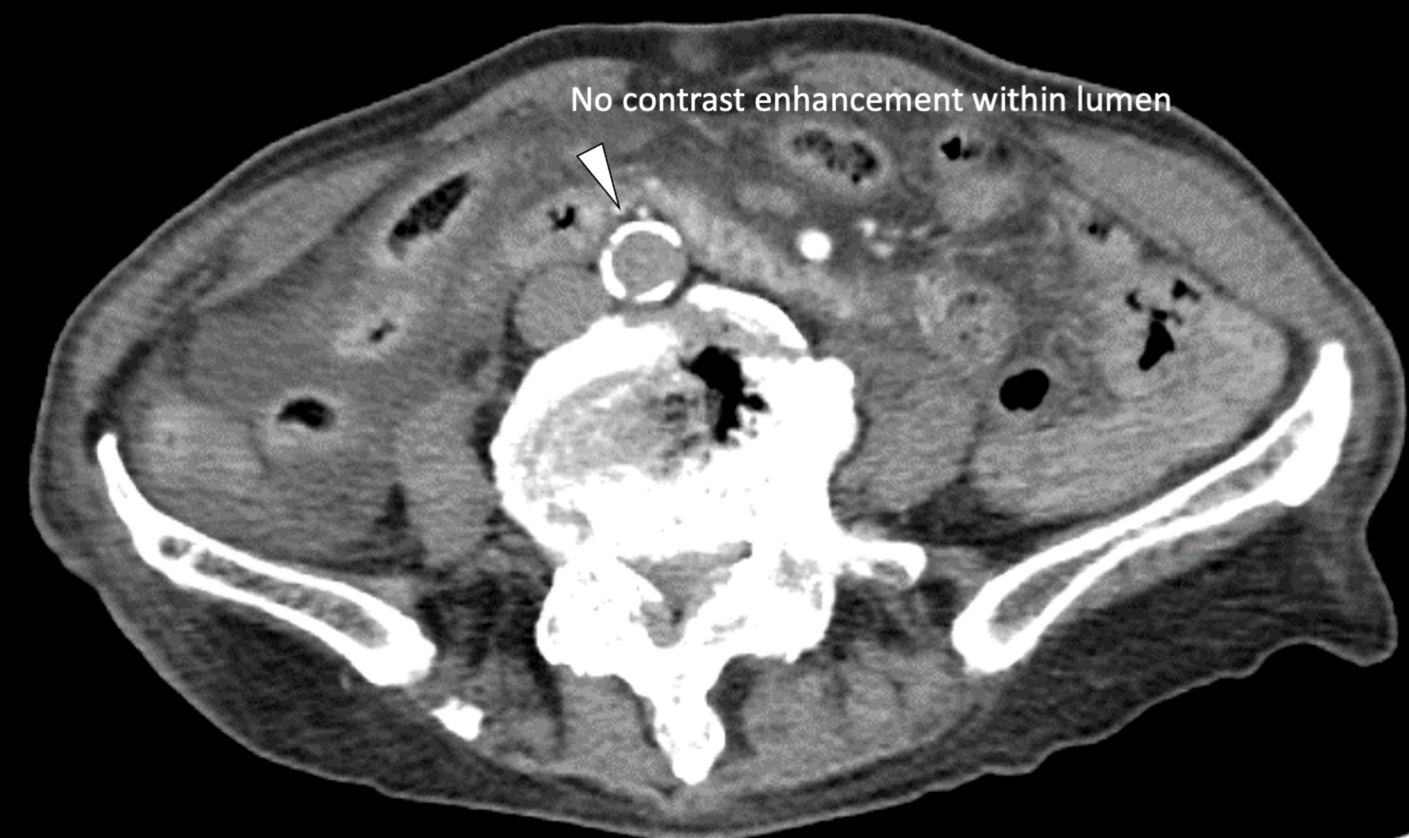
Axial arterial phase CT showing complete distal abdominal aortic occlusion. Axial contrast-enhanced CT image in the arterial phase on day four of hospitalization showing complete thrombotic occlusion of the distal abdominal aorta (arrowhead). The absence of contrast enhancement within the aortic lumen confirms a lack of arterial flow. This finding supports the diagnosis of acute abdominal aortic thrombosis and corresponds with the patient’s acute bilateral lower limb ischemia.

At the time of admission, the patient was hemodynamically stable and had no signs of acute thrombotic or surgical emergency. Therefore, initial management with dexamethasone, remdesivir, and prophylactic heparin was considered feasible at our community hospital. Following initial treatment, oxygen requirements gradually decreased, and the clinical course initially appeared favorable. However, in the early morning of day four, the patient suddenly developed unbearable back pain radiating distally, increased restlessness, and bilateral lower limb paralysis. Physical examination revealed absent bilateral femoral pulses, cold extremities, and loss of deep tendon reflexes. General examination showed no jaundice, cyanosis, or lymphadenopathy. Systemic examination revealed clear heart sounds and no peripheral edema. Emergency contrast-enhanced CT confirmed thrombotic occlusion of the abdominal aorta and absence of distal blood flow, leading to a diagnosis of acute abdominal aortic thrombosis (Figures 2, 3). The patient was urgently transferred to a tertiary care hospital for vascular reconstruction and intensive care, but the subsequent course and final outcome could not be followed.

## Discussion

This case describes a rare presentation of complete acute abdominal aortic occlusion on the fourth day of hospitalization in a 94-year-old male diagnosed with COVID-19, despite the early initiation of antiviral therapy, steroid therapy, and prophylactic anticoagulation. The patient demonstrated markedly elevated D-dimer levels at presentation, indicating an early thrombotic risk. Notably, despite prophylactic anticoagulation, he developed a potentially fatal arterial thrombosis. This case highlights that arterial thrombosis can still occur despite anticoagulation in COVID-19. When acute limb ischemia-like symptoms appear, clinicians must rapidly evaluate for extensive arterial thrombosis, including abdominal aortic occlusion, even if COVID-19 is the primary illness.

Thrombotic events are common in COVID-19, and elevated D-dimer levels are considered a critical marker for risk stratification [1,2]. Viral-induced cytokine storms can trigger endothelial injury and hypercoagulability, promoting thrombus formation in both the venous and arterial circulations [8]. In fact, 10-20% of patients in intensive care settings are reported to experience serious vascular complications, including both venous and arterial thromboses [9].

In this case, the patient’s D-dimer was markedly elevated from admission (53.60 μg/mL), and standard deep vein thrombosis (DVT) prophylaxis with subcutaneous heparin calcium was administered. Although heparin is primarily used for venous thromboembolism prophylaxis, in COVID-19, it also plays an important role in reducing arterial thrombosis risk through antithrombin activation and inhibition of coagulation factors. This is especially relevant in hypercoagulable states, such as those associated with COVID-19 [10]. Nevertheless, the occurrence of extensive arterial thrombosis in this patient suggests that COVID-19-associated coagulopathy may occasionally progress to severe and unmanageable states despite prophylactic anticoagulation.

Similarly, Bellosta et al. reported an increased incidence of acute limb ischemia in COVID-19 patients, including progression of thrombosis despite anticoagulation, highlighting the possibility that the hypercoagulable state associated with COVID-19 may overwhelm standard prophylactic strategies and lead to arterial occlusion even under therapeutic anticoagulation [11]. Baeza et al. described one of the earliest cases of acute aortic thrombosis in a COVID-19 patient, underscoring the potential for large-vessel arterial occlusion despite anticoagulation [5]. More recently, Soumer et al. reported a case series of eight patients with COVID-19 and floating aortic thrombi, most of whom presented with acute limb ischemia and required urgent management [6]. Furthermore, Karabulut et al. conducted a systematic review of 43 reported cases of COVID-19-associated aortic thrombosis, demonstrating substantial morbidity and an overall mortality rate of approximately 30% [7]. Collectively, these studies highlight that COVID-19-related aortic thrombosis can present with diverse clinical scenarios, often with poor outcomes, and emphasize the importance of prompt recognition and individualized management strategies. Although it is possible that anticoagulation in our case may have mitigated the severity to some extent, careful interpretation is needed when evaluating treatment efficacy. Recent studies have debated whether patients with high D-dimer or severe clinical symptoms should receive higher, therapeutic doses of anticoagulation [1,9]. However, therapeutic anticoagulation is closely associated with increased bleeding risk, and the American Society of Hematology (ASH) recommends prophylactic doses for critically ill COVID-19 patients rather than universal therapeutic anticoagulation [12]. Although our patient had a history of arrhythmia, therapeutic anticoagulation was not initiated due to the lack of consensus in current evidence. Whether patients with arrhythmia and markedly elevated D-dimer should receive therapeutic dosing remains uncertain and warrants further investigation. In addition, atrial fibrillation itself is a well-established risk factor for systemic arterial embolism. In this elderly patient, it may have acted synergistically with COVID-19-associated coagulopathy to promote extensive thrombosis. However, given the absence of echocardiographic evaluation or long-term follow-up, its exact contribution in this case remains speculative.

Arterial thrombosis is relatively rare among COVID-19-associated thrombotic events; however, when it occurs, myocardial infarction, ischemic stroke, and acute limb ischemia, which is caused by arterial occlusion, are more commonly reported [3]. Extensive occlusion originating from the abdominal aorta, as seen in this case, is extremely rare. Abdominal aortic occlusion is more commonly caused by trauma, cardiac myxoma, or aortic dissection, and infection-related cases are scarcely reported [13,14]. However, when symptoms such as pain, coldness, or paralysis of the limbs occur, clinicians must avoid misattributing them to non-specific systemic symptoms of COVID-19. Instead, prompt physical examination and contrast-enhanced CT imaging should be pursued to evaluate for vascular obstruction. In elderly patients, symptoms may be atypical; thus, a combination of clinical signs, such as absent femoral pulses, peripheral coldness, and paralysis, can be key to timely diagnosis.

Strengths and limitations

The strength of this study lies in the detailed documentation of a rare presentation of acute abdominal aortic occlusion in a COVID-19 patient, despite prophylactic anticoagulation and early antiviral and steroid therapy. This provides valuable insight into the unpredictable nature of thrombotic complications associated with COVID-19.

However, the main limitation is that this is a single case report, and thus, the findings cannot be generalized. Moreover, we cannot exclude unmeasured confounding factors, such as pre-existing vascular pathology or individual variations in coagulation response. Further studies are required to confirm these observations in larger populations.

## Conclusions

This case describes a rare occurrence of complete acute abdominal aortic occlusion in a 94-year-old man during the acute phase of COVID-19, despite concurrent administration of antiviral, steroid, and anticoagulant therapies. It underscores that severe arterial thrombosis can develop despite prophylactic anticoagulation in COVID-19 patients. When acute limb ischemia-like symptoms appear, clinicians must promptly consider and evaluate for extensive arterial thrombotic events, including abdominal aortic occlusion.

This report reinforces the unpredictable and potentially severe nature of COVID-19-associated thrombosis. It highlights the need for individualized thromboprophylactic strategies in high-risk patients, as well as the importance of accurate physical examination and immediate imaging when atypical symptoms arise in the clinical setting. Nevertheless, as this is a single case report, it cannot dictate changes in standard protocols, and further studies are required to establish optimal management.
